# Probable Enoxaparin-Induced Liver Injury in a Young Patient: A Case Report of a Diagnostic Challenge

**DOI:** 10.7759/cureus.36869

**Published:** 2023-03-29

**Authors:** Ikechukwu E Eze, Sneha Adidam, Domonick K Gordon, Oluwatobi G Lasisi, Jhansi Gajjala

**Affiliations:** 1 Internal Medicine, Howard University Hospital, Washington DC, USA; 2 Family Medicine, Howard University Hospital, Washington DC, USA; 3 Infectious Disease, Howard University Hospital, Washington DC, USA

**Keywords:** low molecular weight heparin, hepatology, adult gastroenterology, liver injury biomarkers, rucam score, drug-induced liver injury (dili), enoxaparin sodium

## Abstract

Low molecular weight heparin (LMWH) is associated with elevated liver enzyme levels in a small percentage of patients. Elevations more than five times the upper limit of normal are uncommon and have been noted to primarily occur in patients receiving higher doses. The literature reports mild, primarily asymptomatic cases, with adverse effects at higher therapeutic doses. We report the case of a 27-year-old woman who developed drug-induced liver injury (DILI) while receiving enoxaparin during admission for a loculated pleural effusion secondary to pulmonary tuberculosis. The Roussel Uclaf Causality Assessment Method (RUCAM) score delineated enoxaparin as the likely cause.

## Introduction

Low-molecular-weight heparin (LMWH) is associated with elevated liver enzymes in only 4-13% of patients [1] and is usually asymptomatic [2]. Elevations higher than five times the upper limit of normal are uncommon and have been noted to occur primarily in patients receiving higher doses [1]. Various resources have been used to establish causation in drug-induced liver injury (DILI); however, no tool has been shown to be clearly superior. We report the case of a 27-year-old woman who developed DILI, likely due to enoxaparin administration during admission for loculated pleural effusion secondary to pulmonary tuberculosis.

## Case presentation

We present the case of a 27-year-old female who presented to our hospital with a three-week history of cough, fever, and weight loss. Her liver enzyme levels were within normal limits on admission. She was started on 40 mg of enoxaparin via the subcutaneous route for thromboembolism prevention. Subcutaneous enoxaparin was temporarily discontinued on day 2 for chest tube insertion to drain pleural effusion. The effusion was exudative with an elevated adenosine deaminase (86.5 U/L) on pleural fluid analysis. Sputum acid-fast bacilli (AFB) smears were positive. On day 8 of admission, first-line antituberculosis medications - rifampin 600 mg daily, isoniazid 300 mg daily, ethambutol 1200 mg daily, and pyrazinamide 1500 mg (RIPE) - were started. Her liver enzyme levels were within the normal range before enoxaparin recommencement on day 10. Elevated liver enzymes were noted four days after enoxaparin recommencement and six days after RIPE was started but continued to increase after the discontinuation of RIPE therapy. The highest liver enzyme levels were noted on day 16 corresponding to six days after restarting enoxaparin. Her liver enzymes at their highest were aspartate transaminase (AST) levels of 875, alanine transaminase (ALT) levels of 489, and alkaline phosphatase (ALP) levels of 188. She subsequently reported nausea and episodes of vomiting. She had no abdominal tenderness or jaundice and no other signs of liver injury. The liver injury pattern was hepatocellular, with an R-factor of 6.1. A medication review by clinical pharmacists revealed no further offending agent. Liver enzymes only subsequently began to decrease with the discontinuation of enoxaparin and promptly reached normal levels within eight days of enoxaparin discontinuation. This corresponds to within 10 days of RIPE therapy discontinuation.

She had no periods of sustained hypotension nor leukocytosis; hemoglobin ranged between 7.2 to 10.2 g/dl all through admission. Her pregnancy test result was negative and she denied alcohol use. The Human Immunodeficiency Virus, Cytomegalovirus, Hepatitis panel, and rheumatoid test results were negative. Abdominal ultrasonography revealed subtly increased echogenicity of the liver with no intrahepatic mass or evidence of biliary tract or gallbladder disease. 

RIPE therapy was restarted without adverse effects or an increase in liver enzyme levels. The patient was diagnosed with a probable symptomatic enoxaparin-induced liver injury. The Roussel Uclaf Causality Assessment Method (RUCAM) score delineated enoxaparin as the probable cause (Table 1) with a RUCAM score of 8. The infectious disease specialist and hepatologist advised holding enoxaparin and anti-tuberculous agents. Of note, RUCAM scoring for anti-tuberculous medications was also calculated and found to be five, which qualifies as a possible cause. Anti-tuberculous agents were re-introduced after liver enzyme normalized, with no effect on liver enzymes. The RUCAM score for the anti-tuberculous agents was 5.

**Table 1:** Hematology/Liver Function Tests WBC: White Blood Cell Count; AST: Aspartate Aminotransferase; ALT: Alanine Aminotransferase; ALP: Alkaline Phosphatase; INR: International Normalized Ratio; APTT: Activated Thromboplastin Time

Tests	On admission	Day 16	Reference range
Hemoglobin	8.8	10.2	12.1 – 15.9 g/dl
Hematocrit	29.1	33.4	34.3 – 46.6%
WBC Count (X10E9)	3.6	4.2	3.2 - 10.9 X 10E9
Platelet (X10E9)	412	418	177 - 406 X 10 E9
AST	18	875	0-50 IU/L
ALT	11	489	0-50 IU/L
ALP	65	188	30 – 130 IU/L
Total Bilirubin	0.5	0.7	0.2 – 1.2 mg/dl
Direct Bilirubin	<0.1	N/A	0.0 – 0.2 mg/dl
Albumin	3.36	2.81	3.2 – 5.5 g/dl
Prothrombin Time	N/A	14.0	25.5 – 35.0 sec
INR	N/A	1.11	1.12 – 1.46
APTT	N/A	34.9	12.5 – 14.5 sec

**Table 2:** Relevant Tests Including Hepatitis Panel, Rheumatology, and Microbiology AFB: Acid-Fast Bacilli; Ab: Antibody; Ag: Antigen; ANA: Antinuclear Antibody; ANCA: Antineutrophillic Cytoplasmic Antibody; HIV: Human Immunodeficiency Virus; CMV: Cytomegalovirus; CCP Ab: Cyclic Citrullinated Peptide Antibody

Test	Result	Reference value
Hepatitis A IgM	Negative	Negative
Hepatitis C Ab	Negative	Negative
Hepatitis B Surface Ab	Negative	Negative
Hepatitis B Core Ab	Negative	Negative
HIV	Non-reactive	Non-reactive
CMV	Negative	Negative
ANA	Negative	Negative
Rheumatoid factor	Negative	Negative
ANCA	Negative	Negative
Legionella Urinary Ag	Negative	Negative
Mycoplasma Pneumonia Ab	Negative	Negative
Blood Culture	Negative	Negative
Urine Culture	Negative	Negative
Sputum AFB	Positive	Negative
Pregnancy Test	Negative	Negative
CCP Ab IgG	Negative	Negative

**Table 3:** Adapted RUCAM Table Criteria for Enoxaparin Versus Anti-Tuberculous Medications in the Index Case Scoring: highly probable > 8 points; probable 6-8 points; possible 3-5 points; unlikely 1-2 points; excluded ≤ 0 points Source: [3]

Criteria	Enoxaparin Score	Anti-tuberculous medication Score	Scoring range
Appropriate temporal relationship (time to onset; latency)	2	1	-1 to +2
Clinical course after drug withdrawal	3	3	−2 to +3
Presence of DILI risk factors (age > 55, alcohol, pregnancy)	0	0	0 to +2
Presence or absence of concomitant hepatotoxic drugs	-1	-1	0 to −3
Search for and exclusion of nondrug causes	2	2	-3 to +2
Prior reports/information confirming the suspect drug's hepatotoxicity	2	2	0 to +2
Response to readministration	0	-2	−2 to +3
Total	8	5	

## Discussion

Drug-induced liver injury (DILI) is a significant cause of liver disease, ranging from asymptomatic liver enzyme elevation to severe liver failure requiring transplantation [3]. Low-molecular-weight heparins (LMWHs) are associated with elevated liver enzyme levels in a small percentage of patients [1]. The mechanism of LMWH-induced liver injury is not entirely understood, but it may be related to hypersensitivity or the direct toxic effects of the drug on hepatocytes [4]. While most cases of LMWH-induced liver injury are mild and asymptomatic, the literature reports mild, primarily asymptomatic, cases with adverse effects at higher therapeutic doses [1]. The case of a 27-year-old woman who developed DILI while receiving prophylactic enoxaparin is a rare instance of a more severe case of LMWH-induced liver injury in a young patient.

RIPE therapy may have contributed to the manifestation of liver injury; it does not seem to be the main culprit in the index case, and consideration for drug-drug interactions seems unfounded. Although the literature has described drug-drug interactions between direct oral anticoagulants (DOACs) and rifampicin, no significant drug interactions have been reported between first-line anti-tuberculous medications and LMWH [5]. The likelihood of causation for anti-tuberculous medication established using the RUCAM score was also lower than that for LMWH. The uneventful reinitiation of all first-line anti-TB agents suggests a reduced role.

The RUCAM score has remained one of the most accessible tools for diagnosing DILI since its introduction in 1993 [6]. It recognizes predisposing factors such as alcoholism, pregnancy, and age greater than 55 years. The index patient did not have any of those predisposing factors. The mean age of patients with DILI is generally reported to be approximately 65 years [7], making our patient an outlier. In this case, the RUCAM score revealed enoxaparin as the likely cause, although anti-tuberculous agents were also identified as a possible cause. However, the index patient had no history of previous exposure to anti-tuberculous agents, and the onset and course of liver enzyme elevation were more consistent with enoxaparin-induced liver injury. Furthermore, liver enzyme levels continued to increase after discontinuation of anti-tuberculous agents but promptly normalized after discontinuation of enoxaparin. Importantly, there was no enoxaparin re-challenge, which clearly contributed to a higher RUCAM score because a further experimental temporal relationship was not established. This is in contrast to the RIPE therapy in this case.

While the RUCAM score is widely used in diagnosing DILI, some debate its fallibility compared to expert opinion or consensus [8]. In our case, a combination of the RUCAM score and expert opinion was used to determine probable causation. Other tools that have been used comparatively to RUCAM include the Revised Electronic Causality Assessment Method (RECAM), which has been observed to align better with expert opinion and has higher reliability for detecting extremes such as a high probability of causation or unlikely causation [6]. However, the current lack of extensive use and validation also remains a drawback. Given its electronic availability, the lack of extensive use and validation may become less of a problem in the near future, as widespread use is likely if it is considered reliable. RECAM is also not used to establish causation when multiple agents are suspected [9]. These limitations may reduce their utility in clinical practice.

Currently, no tool, to the best of our knowledge, has been specifically validated to establish likely causation when more than one drug shows a temporal relationship in the setting of DILI. However, RUCAM accounts for the probability that patients may be on other hepatotoxic agents, making it superior in that regard. The use of RECAM is further limited by its uncertain performance in cases of lower liver enzyme elevation (less than five times the upper limit of normal) [9].

A liver biopsy is the most invasive but accurate method for diagnosing DILI. It can establish the pattern of liver injury and can be a valuable tool for diagnosing DILI. A liver biopsy can also exclude other causes of liver disease. In our case, a liver biopsy was not performed as likely causation was established, and liver enzymes promptly normalized following the withdrawal of the offending agent. Indeed, the decision to perform a liver biopsy must be made on an individual basis, considering factors such as the severity of liver enzyme elevation, the presence of other comorbidities, the value of patient care, and the risks associated with the procedure.

This case exemplifies the clinical complexities of diagnosing DILI.

## Conclusions

LMWH-induced liver injury is rare but can occur even at prophylactic doses. Confounding inpatient factors make it difficult to establish definite causation in many cases of DILI. The RUCAM score, in combination with expert opinion and/or consensus, remains the best tool for delineating the probability and likelihood of DILI causation. A thorough drug history and RUCAM scoring can aid in the diagnosis of DILI and identification of the offending agent while prompt recognition and discontinuation of the offending agent can lead to the resolution of liver enzyme elevations and prevent further liver injury.
